# Efficacy and Safety of Modified Yupingfeng Nasal Spray in Controlling the Recurrence of Persistent and Moderate-Severe Allergic Rhinitis: Study Protocol for a Multicenter, Open-Label, Randomized, and Parallel-Arm Trial

**DOI:** 10.1155/2022/4666332

**Published:** 2022-08-10

**Authors:** Ting Liu, Bing-Qing Lu, Dan-Dan Wang, Chao Liao, Han-Jen Chiang, Rong Zhang, Yuan Xi, Li Tian

**Affiliations:** ^1^Hospital of Chengdu University of Traditional Chinese Medicine, Chengdu 610075, Sichuan, China; ^2^School of Clinical Medicine, Chengdu University of Traditional Chinese Medicine, Chengdu 610075, Sichuan, China

## Abstract

**Background:**

Recurrent episode of allergic rhinitis (AR) is one of the leading illnesses that affects patients. However, there is little research evidence to support pharmacotherapy for AR recurrence. Therefore, this study was designed to explore the efficacy of pharmacotherapy in the control of the recurrence of AR.

**Methods:**

In this study, a multicenter, open-label, randomized, and parallel-arm trial will be conducted at three study centers. A total of 190 subjects aged 18–65 with persistent and moderate-severe AR (Qi deficiency and blood stasis syndrome) will be randomly assigned to receive the modified Yupingfeng nasal spray or mometasone furoate aqueous nasal spray. When subjects' rhinitis control assessment test (RCAT) score is >21 for two weeks, they will stop taking the medication and enter the follow-up. Once a relapse occurs, the time point will be recorded, and the follow-up stops. The primary outcome is the six-month recurrence rate of AR after intervention withdrawal. The secondary outcomes are the one-month recurrence rate of AR, the RCAT score, the duration of follow-up, the duration of medication, the nasal endoscopic results, and questionnaires to evaluate symptoms, signs, and quality of life. The mechanism outcomes include some indicators that may be associated with AR recurrence. In addition, electrocardiograms and other safety indicators will be applied to evaluate the drug's safety. *Discussion*. This is the first study to explore the efficacy of traditional Chinese medicine nasal spray on AR from the perspective of controlling recurrence. The results of this trial may provide valuable clinical evidence for controlling the recurrence of this disease by pharmacotherapy. *Trial Registration*. This study was registered with registration number ChiCTR2100047053 (Chinese Clinical Trial Registry, https://www.chictr.org.cn/showproj.aspx?proj=127432 on June 7, 2021).

## 1. Introduction

Allergic rhinitis (AR) is one of the most common allergic diseases worldwide and it is a global health problem affecting 40% of the world's population [1]. The prevalence of AR has been increasing worldwide since the 1960s [2] and has dramatically increased in recent years [3]. For example, AR showed a sharp increase in prevalence from 11.1% to 17.6% over 6 years in major Chinese cities [4]. The repeated attacks of AR are one of the main difficulties faced by patients. A study of 7-year-olds in South Korea found that the two-year recurrence rate for AR is 72% [5].

The pharmacotherapy of AR includes corticosteroids, antihistamines, and decongestants, most of which focus on suppressing immune factors and controlling immediate symptoms. Even though pharmacotherapy has proven to be highly effective in controlling allergic symptoms, most of the pharmacological management approaches recommended in the guidelines for AR have not been evaluated for efficacy in controlling recurrences. Furthermore, their long-term use can cause side effects, including epistaxis, mucosal dryness, and other adverse reactions, reducing patient compliance. Globally, while the standard pharmacotherapy of AR is relatively mature, the prevalence of AR is still rising [6, 7], and the recurrence rate is still high [8], making it urgent to find new therapies to solve this problem.

The efficacy and safety of traditional Chinese medicine (TCM) have been confirmed in the treatment of AR [9, 10]. According to a meta-analysis, four studies showed that the recurrence rates of AR at three and six months in the TCM groups were significantly lower than those in the control groups [11]. Another meta-analysis reached the same conclusion in children [12]. Yupingfeng powder (YPF) is a classic prescription for treating unsolidified lung-defense Qi syndrome and has shown profound effectiveness in clinical practice in regulating histological morphology, immune factors, and inflammatory cytokines of AR [13, 14] and in treating recurrent respiratory diseases [13, 15, 16]. In TCM, unsolidified lung-defense Qi syndrome is the basic pathogenesis of AR. AR patients generally have a long-term course and high relapse possibility. TCM believes that prolonged illness and repeated attacks of the disease can lead to the formation of blood stasis. In the clinic, symptoms of blood stasis such as narrower diameter, swelling turbinate, and purple and thick veins under the tongue are quite common in AR patients with recurrent attacks. Therefore, Qi deficiency and blood stasis syndrome (QDBS) is a core TCM syndrome of AR recurrence. Based on YPF, modifications have been made to strengthen its function of activating the blood and opening orifices, making it more suitable for AR with recurrent attacks. Although its oral administration is effective, it is not conducive to the rapid relief of AR symptoms due to its limitations, such as slow absorption, easy decomposition by digestive enzymes, and metabolism through the digestive tract and liver [17].

Therefore, the modified YPF has been made into a nasal spray named the modified Yupingfeng nasal spray (MYN), which has the function of “replenishing lung Qi to consolidate exterior and activating blood to open the orifice.” In more than two decades of clinical and animal research, previous studies have shown that MYN can effectively improve the symptoms of AR patients and animal models, reduce the expression of inflammatory factors, regulate immune homeostasis, improve blood stasis, and repair the epithelial barrier of the nasal mucosa [18–23]. Most importantly, we observed that the 4-week follow-up recurrence rate was lower in the MYN group than in the mometasone furoate aqueous nasal spray group [23]. However, that trial was a small, single-center trial with poor quality, which was not conducive to providing high-quality evidence for the efficacy of pharmacotherapy in controlling recurrences of AR. Therefore, a multicenter, open-label, randomized, and parallel-arm trial will be conducted to provide evidence for the efficacy and safety of MYN in the treatment of persistent and moderate-severe AR (QDBS) in the control of recurrence and to explore its possible mechanism.

## 2. Methods

### 2.1. Objectives

The recurrent attacks of AR are one of the main issues that affect patients. However, most of the pharmacological management approaches recommended in the guidelines of AR have not been evaluated for their efficacy in controlling recurrences. Our previous clinical observations showed that MYN achieved several advantages in treating allergic rhinitis, especially in controlling recurrences. Therefore, this study was designed to provide more substantial evidence for the efficacy and safety of MYN in preventing the recurrence of AR.

### 2.2. Trial Design

**Figure 1:**
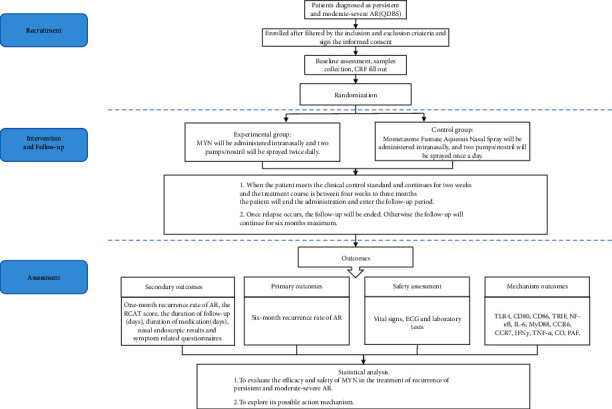
Study flowchart.

We will conduct a multicenter, open-label, randomized, and parallel-arm trial among persistent and moderate-severe AR patients (QDBS), and the ratio of the experimental group to the control group will be 1 : 1. In this study, MYN will be applied to the experimental group, while mometasone furoate aqueous nasal spray will be applied to the control group. The six-month recurrence rate of AR after withdrawing the intervention will be the primary outcome to evaluate the efficacy and safety of MYN in the treatment of recurrence of persistent and moderate-severe AR (QDBS), and this study will also explore its possible action mechanism. We present the protocol in accordance with the Standard Pprotocol Items: Recommendations for Interventional Trials (SPIRIT) reporting checklist (S1). The study flowchart is shown in Figure 1.

### 2.3. Study Setting

Subjects will be enrolled in three comprehensive third-grade first-class hospitals: the Hospital of Chengdu University of Traditional Chinese Medicine, the West China Hospital of Sichuan University, and the Sichuan Provincial Hospital of Integrated Traditional Chinese and Western Medicine (two of them are academic hospitals).

### 2.4. Participants and Recruitment

#### 2.4.1. Diagnostic Criteria of AR

AR belongs to the category of “Biqiu” in TCM [1]. The diagnostic criteria of “Biqiu” will refer to “Otolaryngology of Traditional Chinese Medicine [24].” The Western diagnostic criteria and grading standards for AR will refer to the ‘Chinese Society of Allergy Guidelines for Diagnosis and Treatment of Allergic Rhinitis [1]'. The details are given:

1. Subjects who have typical AR nasal symptoms (nasal itching, rhinorrhea, sneezing, and nasal obstruction) and can have ocular symptoms (ocular grittiness/itching/redness and ocular tearing)
2. Watery nasal discharge, edematous and pale nasal mucosa, and swollen inferior turbinate
3. Positive skin response to house dust mites (HDM) (++ and above) in the skin prick test (SPT)
4. Persistent AR (frequency of symptoms ≥4 days/week and ≥4 weeks/year)
5. Moderate-severe AR (symptoms have a significant impact on quality of life)

#### 2.4.2. Diagnostic Criteria of QDBS

Syndrome element is the minimum diagnostic unit of TCM syndrome diagnosis. QDBS is a combination of “Qi deficiency” and “blood stasis” syndrome elements [25]. According to the diagnostic criteria of “Qi deficiency” and “blood stasis” from the “Guiding Principle for Clinical Study of New Chinese Medicines [26] the syndrome differentiation criteria are given in Table 1. Each center has a Chinese medicine expert who will evaluate TCM syndromes of the participants.

### 2.5. Eligibility Criteria

The inclusion criteria and exclusion criteria are given in Table 2.

### 2.6. Sample Size

In this study, the 6-month recurrence rate was used as the basis for sample size estimation. Combined with our previous research and the literature, the six-month recurrence rates of AR treated with MYN and mometasone furoate aqueous nasal spray were approximately 50% and 75% [27], respectively. Using 5% as the superiority margin, an acceptable alpha of 2.5% (one-tailed), and an acceptable beta of 20%, 86 participants were required in each group. To allow for a 10% loss to follow-up, we will recruit 190 subjects in total.

### 2.7. Recruitment

To ensure adequate participant enrollment, participants will be enrolled in the outpatient department of each center, simultaneously. Participants will also be recruited through advertisements.

### 2.8. Randomization and Blinding

This study adopted a multicenter, open-label, randomized, and parallel-arm controlled design. The randomization method was stratified block randomization. The patients were stratified based on the three different centers, and the ratio of the experimental group to the control group was 1 : 1. According to the given number of seeds and the appropriate length of the segment selected, an independent clinical statistician will use SAS 9.4 statistical software to generate a random sequence and save it as a file in a sealed envelope and then distribute the corresponding envelopes to each center. An independent statistician will keep all of the methods, processes, results, and envelopes of the random sequence. After the beginning of the study, the envelopes will be opened in order of grouping, and recruited subjects will be assigned to treatment groups according to the information in the envelope. Any randomized subject will retain their randomization number after withdrawing from the study for any reason. The random number corresponding to this subject will not be allowed to be reused by other new subjects. Only outcome assessors, examiners, and data analysts will be blinded in this study. Subjects and researchers will not be blinded due to the differences in the color and odor between MYN and the mometasone furoate aqueous nasal spray.

### 2.9. Interventions

#### 2.9.1. Experimental Group

MYN is produced and packed by Sichuan Purity Pharmaceutical Co., Ltd. To ensure the stability of the drug, the same batch of drugs will be used in this study (drug batch number: 051–210917). MYN is currently under patent review. We have uploaded all of the specific components, dosage, and other information of MYN to the official website of China National Intellectual Property Administration, which is open for access and can be found on the patent search and service system page of the official website of China National Intellectual Property Administration [28] via publication no.: CN109908209A. MYN will be administered intranasally and two pumps/nostril will be sprayed twice daily. The drug will not be administered once the patient meets the clinical control standard for two weeks. The shortest treatment time will be not less than four weeks, and the longest treatment time will be not more than three months.

#### 2.9.2. Control Group

Mometasone furoate aqueous nasal spray (NASONEX) is produced and packed by Schering-Plow Labo N. V. (drug batch number: H20140100). Mometasone furoate aqueous nasal spray will be administered intranasally, and two pumps/nostril will be sprayed once a day. The drug will not be administered once the patient meets the clinical control standard for two weeks. The shortest treatment time will be not less than four weeks, and the longest treatment time will be not more than three months.

Any other Western or Chinese medicines for AR are prohibited during this trial.

#### 2.9.3. Clinical Control Standard

After the subjects start treatment, the rhinitis control assessment test (RCAT) will be applied every week for each subject. If the RCAT score is >21, AR is well controlled (clinical control standard); if the RCAT ≤21, it is not controlled.

### 2.10. Outcomes

#### 2.10.1. Baseline Assessment

The demographic characteristics of the subjects (gender, age, current residence, education, and occupation) and general clinical data (medical history, previous treatment history, and AR severity grade) will be collected.

#### 2.10.2. Primary Outcomes

- The primary outcome is the six-month recurrence rate of AR after intervention withdrawal
- Follow-up: subjects will enter the follow-up period after withdrawal of medications. Once a relapse occurs, the patient's recurrence time will be recorded, and the follow-up ends. Otherwise, the follow-up will continue until six months after drug withdrawal.
- Definition of recurrence/relapse: when the RCAT score ≤21 during follow-up, the patient will be considered in a relapse state
- Definition of six-month recurrence rate: six-month recurrence rate = number of recurrent AR patients in six months/total number of patients × 100%
- The RCAT has sufficient reliability, validity, and responsiveness and is a simple patient self-assessment scale [29]. RCAT can be used to assess a patient's rhinitis control status in clinical practice [30]. During the treatment stage, we will collect the RCAT scale online once a week, and during the follow-up stage, we will collect the RCAT scale online every two weeks.

#### 2.10.3. Secondary Outcomes

The secondary outcomes are the one-month recurrence rate of AR, the RCAT score, the duration of follow-up (days), the duration of medication (days), the nasal endoscopic results, the visual analog scale (VAS), the total nasal symptom score (TNSS), the rhinoconjunctivitis quality of life questionnaire (RQLQ), the total ocular symptom score (TOSS), the TCM syndrome score of spleen Qi deficiency and lung Qi deficiency, and the classification and determination scale of constitution of TCM.

(1) $$\begin{array}{c} \text{Definition of one}-\text{month recurrence rate}=\frac{\text{Number of recurrent AR patients in one month}}{\text{Total number of patients}}\times 100\%. \end{array}$$

All of these will be recorded before/after the intervention and when relapse occurs on the paper version of the CRF.

#### 2.10.4. Mechanism Outcomes

- Serum: endogenous carbon monoxide in serum will be measured by colorimetry. PAF will be measured by quantitative real-time PCR (qRT-PCR). The expression levels of TLR4, CD80, CD86, CCR6, and CCR7 on the surface of DCS will be detected by flow cytometry (FCM).
- Cells: epithelial nasal mucosa cells will be extracted with a nasal brush. The mRNA and protein expression levels of TLR4, MyD88, and TRIF in the nasal mucosa will be measured by qRT-PCR and Western blotting, respectively. The protein expression levels of HO-1 and TLR2 will be measured by Western blot.
- Nasal lavage fluid: the nasal lavage fluid concentrations of IL4, IFN*γ*, TNF-*α*, NF-*κ*Β, IL-6, and IL-12 will be measured by ELISA.
- Not all subjects need to participate in the collection of mechanism samples. A subgroup of 18 subjects will be asked to participate in an optional study, in which the epithelial cells of the nasal mucosa will be extracted with a nasal brush, and the serum and nasal lavage fluid will be collected to measure the mechanism indicators. These samples will be collected at baseline and after drug withdrawal. Regardless of treatment allocation, subjects can voluntarily participate in this part of the trial through instructions provided on the informed consent form. To ensure a consistent baseline, we will use a random number table to randomly select nine willing subjects from each of the experimental and control groups.

#### 2.10.5. Safety Assessment

Vital signs, ECG, and laboratory tests (liver and kidney function and blood and urine routine examination) will be compared before and after interventions, and the safety of the interventions will be assessed when AR is controlled. The subjects should contact the researchers if any side effects or adverse events occur. After receiving the patient's feedback, investigators will inform the subjects to visit the hospital for further assessment. In addition, a urine pregnancy test should be performed before female subjects of childbearing age are recruited.

### 2.11. Participant Timeline

Participants' timeline is given in Table 3.

### 2.12. Statistical Methods

All analyses will be based on the intention-to-treat (ITT) principle. Missing data will be reduced to a minimum. For data loss caused by subject drop-out, data from the previous measurement will be carried forward. The frequency, percentages, means and standard deviations, medians, and quartiles will be calculated for descriptive analysis. Intergroup comparisons of continuous variables will be performed using the *t*-test or Mann–Whitney *U* test. Categorical variables will be compared using Pearson *χ*^2^ tests or Fisher exact tests. Time-to-event outcomes will be analyzed using the Kaplan–Meier method and Cox proportional hazard models. Two-tailed *p* < 0.05 will be considered statistically significant for all analyses. All statistical analyses will be conducted using SPSS version 25.0 for Windows (IBM Corporation, Armonk, NY, USA).

### 2.13. Strategies to Improve Adherence to Interventions and Promote Participant Retention and Complete Follow-Up

High compliance consists of adequate communication with the subjects, providing appropriate reward mechanisms and establishing a good doctor-patient relationship. During recruitment, the subjects will receive extensive information about the study. Subjects will receive free treatment and examinations and will be correspondingly compensated after each follow-up visit to our clinic. Researchers will emphasize the importance and benefits of completing the follow-up. Because AR is a recurrent disease, subjects may relapse after a period of remission. This study will follow the whole process of AR of subjects from onset to clinical control to recurrence and provide corresponding clinical advice to the subjects in case of recurrence. Moreover, a medication compliance index will be used to evaluate the subjects' compliance.

### 2.14. Data Management and Monitoring

Data entry and management are the responsibility of dedicated data managers. Scales will be collected through CRF. Before recruitment, all researchers worked together to formulate the standard operating procedure (SOP) of this study. According to the SOP, the principal investigator (PI) regularly holds training meetings for all researchers to guarantee interobserver consistency to ensure the reliability of clinical data. A quality control monitor will confirm that the records and reports of all research data are correct and complete and ensure that they are consistent with the original data throughout the study.

### 2.15. Criteria for Adverse Event Reporting and Discontinuing or Modifying the Allocated Interventions

Subjects may refuse to participate in the study or withdraw from the study at any time during the study without affecting their medical treatment and benefits. If a patient is uncooperative, the investigators can also end the patient's participation in this study. The investigators will make a follow-up appointment (by phone, e-mail, and message), contact the subject if possible, inquire about the reason for withdrawing, the time of last medication, and complete as many assessment items as possible. If any adverse event (AE) occurs during the trial, the responsible investigator should take the necessary measures to provide treatment. Any serious adverse events (SAEs) will be reported within 24 h to the sponsor and the ethics committee. The manifestation, severity, onset time, duration, and prognosis of the adverse events will be observed and recorded in detail. A committee of medical experts will determine whether it is related to the treatment. In the case of AE and injuries caused by diagnostic tests or research drugs, effective measures should be taken to ensure the safety of the subjects. Moreover, the research team will pay the related treatment costs and the corresponding economic compensation according to China Good Clinical Practice Guideline.

### 2.16. Confidentiality

All study-related information (basic information, data, laboratory reports, forms, and informed consent) will be identified by the random sequence number and stored by the PI in lockers or encrypted on designated computers. Serum, nasal lavage fluid, and nasal mucosa samples from this study will be placed in a locked refrigerator. Data containing personal identifiers will be destroyed three years after the end of the trial.

### 2.17. Ethics and Dissemination

This study will be conducted under the guidance of the Helsinki Declaration and has been approved by the ethics committees of all medical centers (Table 4) (S2). If it is necessary to modify the protocol, we will submit the modified protocol to the funder and the ethics committees of this study. After obtaining the approval of the funder and the ethics committees, we will revise the protocol and record it on the Chinese Clinical Trial Registry. The complete protocol for this study is available on the official website of the Chinese Clinical Trial Registry [31] (registration number: ChiCTR2100047053). The results will be presented to subjects by telephone after the follow-up period. Moreover, the results will be reported according to the Consolidated Standards of Reporting Trials (CONSORT) in medical journals and presented at academic conferences to benefit researchers, clinicians, and patients.

## 3. Discussion

Mometasone furoate aqueous nasal spray will be administered to the control group in this study. Intranasal corticosteroids (INCs) are first-line treatments for persistent or moderate-severe AR patients [32]. INCs are effective for the four significant classic symptoms of AR and ocular symptoms of AR [1]. INCs are more effective than H1-antihistamines and leukotriene receptor antagonists, especially for nasal obstruction [32]. The mechanism of action is related to the local anti-inflammatory effect of nasal mucosal cells. Second-generation INCs further minimize systemic bioavailability (<1%) compared with first-generation INCs and oral corticosteroids [33], thereby limiting systemic effects on AR. Some studies have shown that AR is a local reaction of systemic inflammation and systemic immune inflammation of multiple organs and systems caused by allergen invasion [34]. The existence of systemic inflammation will keep the body in a sensitized state, and it is easily restimulated when encountering allergens, resulting in recurrence. Therefore, the insufficient systemic efficacy of INCs also limits their efficacy in controlling recurrences.

In contrast, the comprehensive regulation of multitargets and multilinks is a characteristic of TCM [35]. The TCM theory holds that “the lung opens into the nasal.” On the one hand, nasal disease is the external manifestation of lung status. On the other hand, the nose communicates directly with the lung, and drug administration from the nose can directly affect the lung. It should be emphasized that the concept of “lung” in TCM is not the concept of the anatomical lung but a high generalization of a series of “lung” functions. Moreover, maintaining the body's defense function is one of the lungs' primary functions. Therefore, through nasal administration, the medication can directly affect the lung, and the filled lung Qi can regulate the defense function of the nasal cavity and the whole body; in this way, the recurrence of AR can be controlled. Although INCs and MYN are both administered intranasally and can control the symptoms of AR, their underlying action logic is different, and we believe that this difference is the key to controlling recurrences. Furthermore, compared to other kinds of nasal corticosteroids, the mometasone furoate aqueous nasal spray is safer and can be administered to children above three years. Given the reasons mentioned above, we chose the mometasone furoate aqueous nasal spray as the intervention in the control group.

Subjects between 18 and 65 will be enrolled in this study. All of the herbs in MYN are herbs with high safety. Moreover, MYN has been used in the clinic for more than 20 years, and the efficacy and safety of MYN have been approved [18–23]. The safety of the mometasone furoate aqueous nasal spray is internationally recognized and can be administered to children above three years old [1,33]. This study will recruit AR subjects aged 18–65 years because most Chinese children under 18 years, especially those aged 13–18 years, are junior and senior high school students who live on campus, and it is difficult to require timely follow-up visits during the follow-up period. Therefore, people in this age group were not included in the study. The elderly population (over 65 years) has a distinct clinical presentation, including an increased incidence of rhinorrhea symptoms and a low prevalence of AR [36]. The management of elderly AR patients may differ from that of the general adult population [32]. Therefore, this age group was not included in the study.

According to previous research [21], symptom scores returned to normal on the 14th day of MYN administration in animal models. The short-term treatment course of nasal corticosteroids for AR is 2–12 weeks [37]. This study will only enroll patients with persistent and moderate-severe AR, and the short-term treatment course of nasal corticosteroids for moderate-severe AR is four weeks. Therefore, the shortest treatment course should not be less than four weeks, and the longest treatment should not be more than three months. Subjects should continue the treatment for two weeks until the clinical control standard has reached a stable effective state.

The RCAT scale was used to determine whether patients experienced AR relapse. There is no “gold standard” for evaluating the efficacy of AR [38]. The RCAT is one of the most widely validated rhinitis control scales, and its reliability, validity, and responsiveness have been widely validated [38]. The RCAT is a simple, self-rating scale that allows patients to assess their AR control at home [30] and helps gauge the success of therapeutic interventions repeatedly over a long period [29, 39]. Therefore, in this study, the RCAT will be regularly sent to subjects, and the RCAT score will be recorded to reflect the rhinitis control status and relapse status of subjects. In this study, the six-month recurrence rate of AR after drug withdrawal will be the primary outcome, reflecting the long-term efficacy of MYN in controlling relapses. The one-month recurrence rate will be used to reflect the short-term efficacy of MYN in controlling AR relapses. This will directly reflect the speed of relapse through the duration of follow-up. The longer the follow-up is, the slower it recurs; conversely, the shorter the follow-up is, the faster it recurs. In fact, more meaningful data would have been collected if follow-up had been extended. However, considering that the loss rate of subjects will increase with the extension of follow-up time, prolonged follow-up time may have many uncertainties affecting the accuracy of the study results. Therefore, the maximum follow-up time of this study is 6 months. If the results of this experiment show that MYN can control the recurrence of AR, the follow-up period will be extended to observe the efficacy of prolonged control of recurrence in future studies.

Limitations: this study also has limitations. For example, MYN is a nasal spray made from pure Chinese medicine. It is brown and has a light herbal odor, while the mometasone furoate aqueous nasal spray is colorless and odorless, so it is difficult to blind subjects to treatment. Therefore, this study adopts an open-label design, but this may cause bias in evaluating the efficacy of relapse control. Nasal endoscopy examination will be conducted on subjects before and after interventions to objectively reflect the efficacy of the interventions and minimize the influence of psychological effects on subjects in an open-label design. Efforts to make MYN colorless and odorless are ongoing.

## 4. Conclusion

Globally, the prevalence of AR is still rising, and the recurrence rate is still high, while the standard pharmacotherapy of AR fails to focus on reducing recurrences, making it urgent to find new therapies to solve this problem. Thus, this study may provide evidence for the efficacy of pharmacotherapy of AR recurrence by TCM and provide more options for the treatment of AR in clinics.

### 4.1. Trial Status

The recruitment started on September 1, 2021, and is expected to finish on December 31, 2022, 16 months in total.

## Figures and Tables

**Table 1:** Syndrome differentiation criteria of QDBS.

	Qi deficiency	Blood stasis
Primary symptoms	Shortness of breath; mental fatigue; and feeble pulse	Obstruction of collaterals manifestations (purplish lips, gingiva, and nails); purplish tongue or tongue with ecchymosis; varicose veins under the tongue; and unsmooth pulse/deep and wiry pulse/slow and wiry pulse.

Secondary symptoms	Spontaneous sweating; unwillingness to speak; and pale tongue	Scaly skin; hypoesthesia; depressive; amnesia; and paresthesia.

*Note*. Diagnosis of each syndrome element should include two primary symptoms or one primary symptom and two secondary symptoms, simultaneously. The diagnosis of QDBS requires meeting both diagnoses of “Qi deficiency” and “blood stasis,” simultaneously.

**Table 2:** Study inclusion and exclusion criteria.

Inclusion criteria	• Patients who meet the above diagnostic standards
	• Patients with a positive skin prick test (SPT) of HDM of grade 2 or above
	• Patients between 18 and 65 years regardless of gender
	• Patients who have not received any medication for AR in the past two weeks
	• Patients who agree to participate in this study and sign the informed consent form (S3)

Exclusion criteria	• Patients combined with severe nasal septal deviation, chronic nasal-sinusitis, bronchial asthma, nasal polyps, and other diseases
	• Patients with heart, liver, and kidney disorders or autoimmune diseases
	• Patients with mental illnesses, mental disorders, and/or unable to cooperate well enough to complete the study
	• Patients during pregnancy, lactation, or pregnancy preparation
	• Patients who are allergic to the composition of the trial drugs
	• Patients who have received glucocorticoids, leukotriene receptor blockers, antihistamines, or anti-inflammatory drugs within the past two weeks
	• Patients with skin pathological changes at the site of the skin prick test
	• Patients who have previously participated in specific immunotherapy
	• Patients who have participated in other clinical studies in the past two months. Patients with any one of the above shall be excluded.

**Table 3:** Participants' timeline of this study.

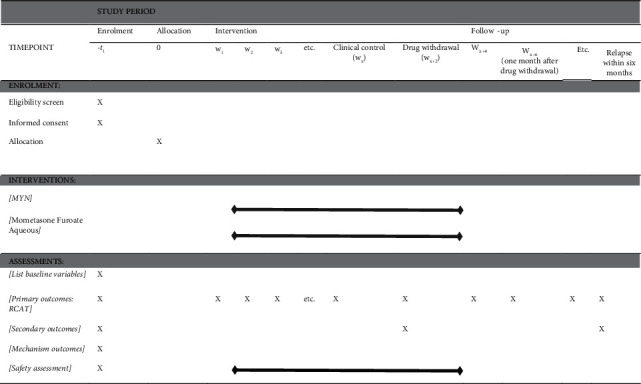

*Note*. w, week; w1, the first week; w2, the second week; Wx, timepoint when subjects meet the clinical control standard; drug withdrawal (wx+2), timepoint when subjects meet the clinical control standard and last for two weeks.

**Table 4:** Ethical review approvals.

Number	Ethics committees' name	Approval registration number
1	Medical Ethics Committee of the Hospital of Chengdu University of Traditional Chinese Medicine	2021KL-046
2	Ethics Committee on Biomedical Research, West China Hospital of Sichuan University	n/a
3	Medical Ethics Committee of the Sichuan Provincial Hospital of Integrated Traditional Chinese and Western Medicine	KY-HX-2021-039

## Data Availability

Data sharing is not applicable to this article as no datasets were generated or analyzed during the current study. The data will be shared after the trial is finished.

## Abbreviations

AR: Allergic rhinitis
TCM: Traditional Chinese medicine
YPF: Yupingfeng powder
MYN: Modified Yupingfeng nasal spray
QDBS: Qi deficiency and blood stasis syndrome
RCAT: Rhinitis control assessment test
VAS: Visual analog scale
RQLQ: Rhinoconjunctivitis quality of life questionnaire
TNSS: Total nasal symptom score
TOSS: Total ocular symptom score
qRT-PCR: Quantitative real-time PCR
FCM: Flow cytometry
ECG: Electrocardiogram
AIT: Allergen-specific immunotherapy
SPT: Skin prick test
SOP: Standard operating procedure
PI: Principal investigator
ITT: Intention-to-treat
INCs: Intranasal corticosteroids.

## Supplementary Materials

Supplementary Materials S1, SPIRIT checklist.PDF; S2, ethical approvals.docx; S3, informed consent (supplementary materials).
