# Supplementary material for: Efficacy and Safety of Modified Yupingfeng Nasal Spray in Controlling the Recurrence of Persistent and Moderate-Severe Allergic Rhinitis: Study Protocol for a Multicenter, Open-Label, Randomized, and Parallel-Arm Trial
Source: Evid Based Complement Alternat Med. 2022 Aug 10;2022:4666332. doi: 10.1155/2022/4666332 (PMC9385272; doi:10.1155/2022/4666332)

**Medical Ethics Committee of Hospital of Chengdu University of Traditional Chinese Medicine**

**Ethical review approval**

| **Ethical approval number** | **2021KL-046** | | |  |
| --- | --- | --- | --- | --- |
| **Project name** | Efficacy of Modified Yupingfeng Nasal Spray in Controlling the Recurrence of Persistent and Moderate-Severe Allergic Rhinitis: Study Protocol for a Multicentre, Open-Label, Randomized, and Parallel-Arm Trial | | |  |
| **Funding** | Science &Technology Department of Sichuan Province(P.R. China) | | |  |
| **Clinical research institution** | Hospital of Chengdu University of Traditional Chinese Medicine, West China Hospital of Sichuan University, Sichuan Provincial Hospital of Integrated Traditional Chinese and Western Medicine | | |  |
| **Principal investigator of this centre** | Li Tian | | | |
| **Category of the review** | Review | **Method of review** | Quick review | |
| **Review date** | 2021-05-28 | **Review site** | Office of Ethics Committee | |
| **Approval documents of the review** | 1. Application for review 2. Clinical Research Protocol (20210521, V1.4) 3. Informed Consent (20210521, V1.4) 4. Subject recruitment materials (20210510, V1.3) 5. Research records and/or case report form, the subject's diary card, and other questionnaires (20210421, v1.1) | | | |
| **Review opinions** | | | | |
| According to the 2020 *Quality Management Practice for Drug Clinical Trials, the 2016 Medical Equipment Quality Control Standard for Clinical Trials, and the Guiding Principles for Ethical Review of Drug Clinical Trials* issued and implemented by CFDA, the *Approach to the Ethical Review of Biomedical Research involving People* issued and implemented by national health commission of the people’s republic of China in 2016, *the Chinese Medicine Clinical Research Ethics Review Management Norms* issued and implemented by national administration of traditional Chinese medicine in 2010, the *Helsinki Declaration* and the *International Ethical Guidelines for Human Biomedical Research*, etc. After the review of our ethics committee, it is agreed to carry out the clinical study according to the approved documents.   1. Please follow the GCP principles and initiate the clinical study as soon as possible, following the protocol approved by the ethics committee. 2. Particular attention should be paid to: 3. Serious adverse events and critical medical events that must be reported according to the protocol, please submit corresponding reports; (2) For any modification of clinical study protocol, informed consent, recruitment materials, and change of principal investigator, please submit the application form for amendment review; (3) The principal investigator should submit a research progress report within 1 month prior to the due date in accordance with the annual/regular follow-up review frequency prescribed by the Ethics committee; The sponsor shall submit a summary report on the research progress of each center to the ethics Committee of the group leader; （4）The sponsor or principal investigator is requested to submit a protocol violation report if any of the following occurs： Subjects who did not meet the inclusion criteria or met the exclusion criteria were included in the study；Did not withdraw a subject from the study who met the discontinuation requirements；To give the wrong treatment or dose；The administration of drug combinations prohibited by the protocol and the failure to follow the protocol；Any violation of GCP principles that may adversely affect the rights/health of the subjects and the science of the research；(5) If the sponsor suspends/terminates the clinical study in advance, please submit the report of suspension/termination in time and restart the suspended study. The applicant should apply for ethical approval through the "Research Progress Report";(6)The primary investigator shall submit a final report after completing the clinical study.   3. For research involving human genetic resources, please strictly comply with the relevant provisions of the Regulations on the Management of Human Genetic resources, timely complete the declaration and filing to the China Human Genetic Resources Management Office, and start the research only after obtaining the approval. | | | | |
| **The validity period of this approval** | 2021.05.28-2024.05.27 | **Frequency of follow-up review** | 12 months | |
| **Contact person and contact information** | Qing He: 86-028-87783142, ethicscd@126.com | | | |
| **Chairman's signature** | Chengshi He | | | |
| Medical Ethics Committee of Hospital of Chengdu University of Traditional Chinese Medicine  （seal） | | | | |
| 28 May 2021 | | | | |


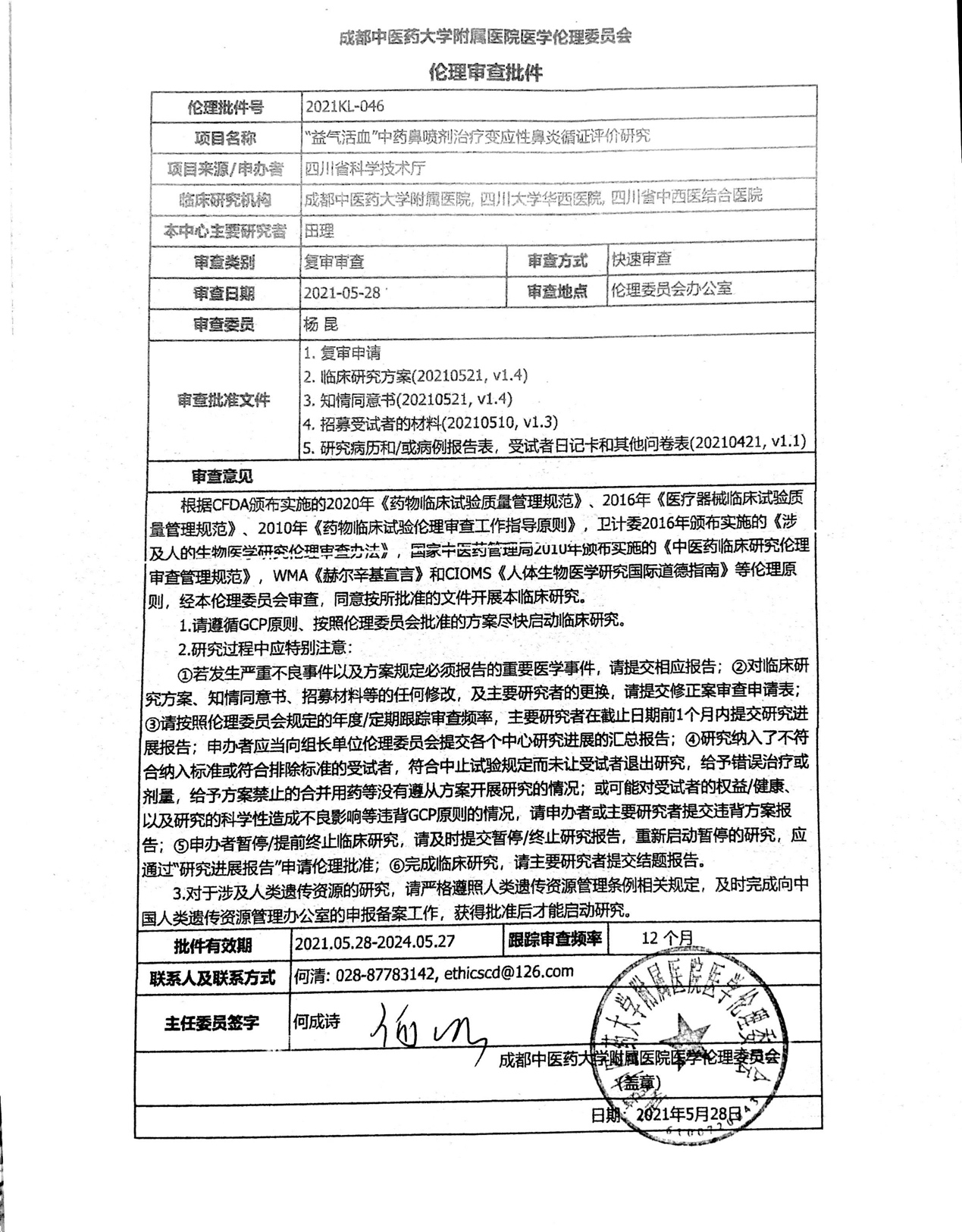


Ethics Committee on Biomedical Research, West China Hospital of Sichuan University

Ethical approval documents

2021 Review No. (1206)

| **Department:** Department of Otolaryngology head and Neck Surgery | | Name and title of principal investigator in this centre: Juan Meng /Associate professor | |
| --- | --- | --- | --- |
| **Project Name** | Efficacy of Modified Yupingfeng Nasal Spray in Controlling the Recurrence of Persistent and Moderate-Severe Allergic Rhinitis: Study Protocol for a Multicentre, Open-Label, Randomized, and Parallel-Arm Trial | | |
| **Study protocol** | Version:V1.6 | | Date:26 December 2021 |
| **Informed consent** | Version:V1.6 | | Date:26 December 2021 |
| **Recruitment advertisement** | Version:V1.6 | | Date:26 December 2021 |
| **Review opinions:**  1. The qualifications of researchers meet ethical requirements.  2. The study protocol and informed consent met the ethical requirements.  3. Recruitment advertisement meet ethical requirements.  **Results of ethical review：Approval**  **Frequency of ongoing review：once a year**  Please follow relevant laws, regulations and rules of China (‘Ethical Review Measures for Biomedical Research Involving People’, etc.) and The WMA ‘Declaration of Helsinki’ and CIOMS ‘International Ethical Guidelines for Human Biomedical Research’ protect the health and rights of subjects by conducting clinical trials (studies) in accordance with protocols and informed consent approved by ethical review boards.  Please strictly implement the ‘regulations on the administration of human genetic resources of the People's Republic of China’ (with no. 717), concerning our human genetic resources collection, preservation, international cooperation, material exit all needs to apply to the national Ministry of Science and Technology, the action of administrative license, then you can use the information available or open to the national Ministry of Science and Technology application backup for the record, and through the rear can implement the corresponding activities. The Department of Clinical Research Management, as the department of human genetic resources management of the hospital, tel: 85422851.  In the course of the trial (study), if the principal investigator is changed, any modification to the clinical study protocol, informed consent, etc.. Applicants are invited to submit an amendment review application.  If a serious adverse event occurs, the applicant should submit a serious adverse event report in time; Submit details as soon as possible after the emergency report. Follow-up reports of serious adverse events were reported.  Please submit annual and periodic follow-up review reports; Applicants are requested to submit written reports to the IRB in a timely manner in the event of any situation that may significantly affect the conduct of the study or increase the risk to the subject.  The study did not comply with the protocol by including subjects who did not meet the inclusion criteria or the exclusion criteria, failing to withdraw subjects from the study in accordance with the suspension criteria, giving wrong treatment or dose, giving drug combinations prohibited by the protocol, etc.; Or the violation of ethical principles and norms, which may have adverse effects on the rights and interests/health of the subjects, as well as the scientific nature of the study, shall be submitted by the sponsor/supervisor/researcher.  If the applicant suspends or discontinues the clinical trial (study) in advance, please submit the suspension/termination report in time. To complete the clinical trial (study), please submit the final report.  Clinical research cannot be carried out without ethical review and approval.  This approval is valid for one year, failing to implement within the time limit, it will be abolished automatically.  According to the International Board of Editors of Medical Journals (ICMJE), all clinical studies conducted in and from human specimens should be registered. The investigator who has received the ethical approval must register at the China Clinical Research Registry before the clinical study begins. Please log on to the following website for clinical study registration using the public account of our hospital (please apply by email to the Department of Clinical Research Management hxlcyjglb@163.com, contact number: 85422851) : HTP :// w.chictr.org.cn The unique registration number generated after the successful registration of the clinical research project, please send an email to the Ethics office (huaxilunli@163.com) in time. It is a required item of the ethics follow-up review  Institution(seal)  Chairman's signature | | | |


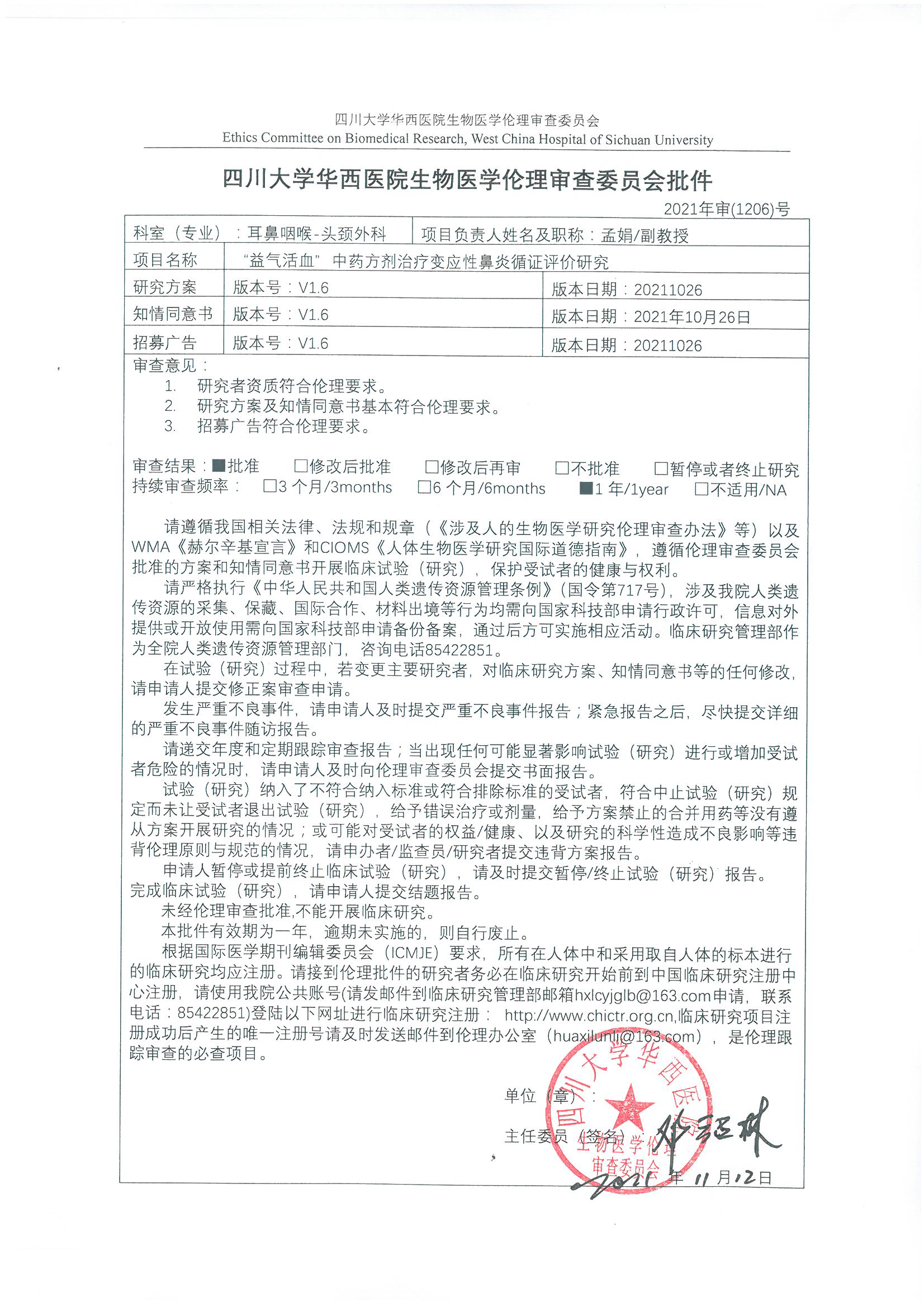


Medical Ethics Committee of the Sichuan Provincial Hospital of Integrated Traditional Chinese and Western Medicine

Ethical approval documents

| **Ethical approval number** | | KY-HX-2021-039 | | | |
| --- | --- | --- | --- | --- | --- |
| **Project name** | | Efficacy of Modified Yupingfeng Nasal Spray in Controlling the Recurrence of Persistent and Moderate-Severe Allergic Rhinitis: Study Protocol for a Multicentre, Open-Label, Randomized, and Parallel-Arm Trial | | | |
| **Sponsor** | | Hospital of Chengdu University of Traditional Chinese Medicine | | | |
| **Clinical research institution** | | Sichuan Provincial Hospital of Integrated Traditional Chinese and Western Medicine | | | |
| **Principal investigator of this centre** | | Xiao-Juan Wu | | | |
| **Review date** | 10 June 2021 | **Review site** | | Office of Ethics Committee | |
| **Approval documents of the review** | | Application for review  Clinical Research Protocol  Informed Consent  Research records and/or case report form, the subject's  Major investigator's professional resume  List of investigators in clinical trials | | | |
| **Member of the review Committee** | | Zu-Bo Huang, Cong-Cong Yu, Li-Ping Zhou, Shu-Tao Chen, Mao-Xin Yu, Ju Wen, Chen Li, Sheng-Xia Xiang, He Ai, Zai-Yang Zhu, Jin-Yan Yang | | | |
| **Review opinions** | | According to the state food and drug administration of the People's Republic of China promulgated in 2003 implemented the quality control standard for clinical trials, enacted in 2010, the guiding principles in the drug test bed ethical review, 2016 In the national health committee relating to the people of biomedical research ethics review method ", the world medical association declaration of Helsinki and international medical science organization committee issued "human body biomedical research international morality refers to the south" ethical principles, such as approved by the ethics committee review, agree to carry out this research project.  Please follow the GCP principles and follow the protocol approved by the ETHICS committee. In the process of the research should pay special attention to protect the health and rights and interests of the subjects, in case of serious adverse event and an unexpected adverse events influenced the risk benefit ratio, please submit adverse event reports; To study scheme, informed consent, the recruitment materials, etc. Any modification, and mainly research the change, please submit an application for amendment to review report; The applicant suspend/terminate study, please timely submit to suspend/terminate study report; Complete the study, please submit a problem report. | | | |
| **The validity period of this approval** | | Two Years | **Frequency of follow-up review** | | One Year |
| **Contact person and contact information** | | Hao Zhou: 028-65357112 | | | |
| **Chairman's signature** | | Zu-Bo Huang | | | |
| Medical Ethics Committee of the Sichuan Provincial Hospital of Integrated Traditional Chinese and Western Medicine(seal) | | | | | |
| 10 June 2021 | | | | | |


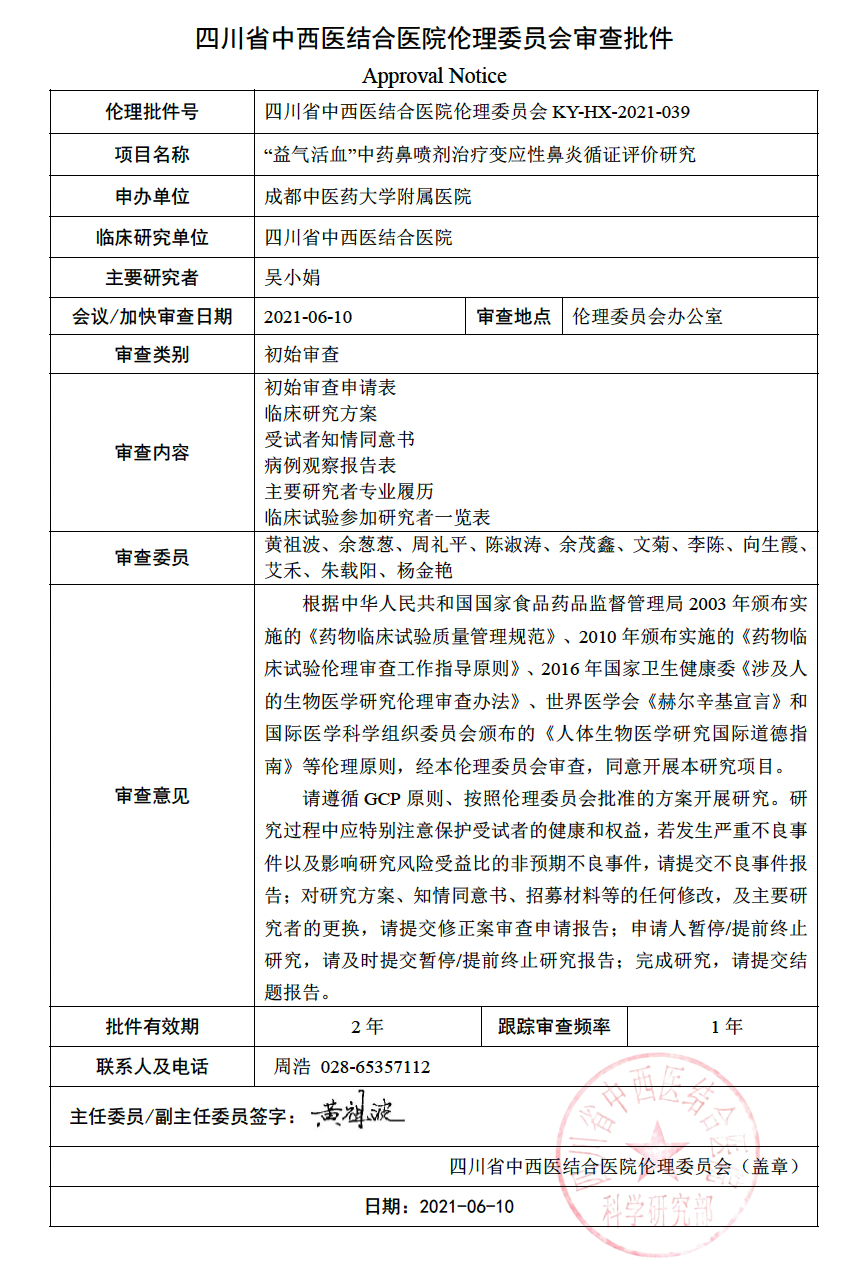

Supplement: Supplementary Materials — S1, SPIRIT checklist.PDF; S2, ethical approvals.docx; S3, informed consent (supplementary materials). [file 4666332.f1.zip › S2 Ethical approvals.docx]
