# Supplementary material for: Efficacy and Safety of Modified Yupingfeng Nasal Spray in Controlling the Recurrence of Persistent and Moderate-Severe Allergic Rhinitis: Study Protocol for a Multicenter, Open-Label, Randomized, and Parallel-Arm Trial
Source: Evid Based Complement Alternat Med. 2022 Aug 10;2022:4666332. doi: 10.1155/2022/4666332 (PMC9385272; doi:10.1155/2022/4666332)
Supplement: Supplementary Materials — S1, SPIRIT checklist.PDF; S2, ethical approvals.docx; S3, informed consent (supplementary materials). [file 4666332.f1.zip › S3 Informed consent.doc]

# Informed consent

**Project name：** Efficacy and safety of Modified Yupingfeng Nasal Spray in Controlling the Recurrence of Persistent and Moderate-Severe Allergic Rhinitis: a multicenter, open-label, randomized, and parallel controlled trial

**Name of research Center：** The Hospital of Chengdu University of Traditional Chinese Medicine, the West China Hospital of Sichuan University, and the Sichuan Provincial Hospital of Integrated Traditional Chinese and Western Medicine

——————————————————————————————————

Dear patient：

The doctor has preliminary diagnosed you with allergic rhinitis. Now we invite you to participate in a clinical study entitled “Efficacy of modified Yupingfeng nasal spray(MYN) in controlling recurrence of persistent moderate-severe allergic rhinitis: a multicenter, open-label, randomized, and parallel controlled trial."

**Firstly,** you need to understand that participation in this clinical study is entirely voluntary.

**Secondly, t**here is a big difference between the treatment you receive in a clinical study and the treatment you receive from your doctor outside the study, and the research team needs to treat you following the clinical study protocol

requirements.

The Science and Technology Department has approved the project of Sichuan Province, and the Medical Ethics Committee of the Hospital of Chengdu University of Traditional Chinese Medicine has conducted the ethical review and approved it. In addition, the project has also been registered on the Chinese Clinical Trial Registry. Registration Website.

I. **Research background**

Allergic rhinitis is one of the most common allergic diseases worldwide, a global health problem affecting 40% of the world's population. Allergic rhinitis, got a sharp increase in prevalence from 11.1% to 17.6% in 6 years, was estimated to cost about 14.79 billion dollars per year in China. The therapeutic drugs recommended by many domestic and foreign guidelines are divided into two types: oral and nasal to form a standard AR diagnosis and treatment system. Oral medications, such as antihistamine and anti leukotriene, have limited effect with a single target. Due to the features of no cuticle and full of capillaries in the nasal mucosa, drugs can directly affect the lesions, endowed it with the advantages of ease of administration, rapid onset of action, and avoidance of the first-pass metabolism. International AR guidelines have included nasal corticosteroids as AR recommendations since 2001. Nasal corticosteroids are one of the most effective medications for the treatment of AR. Its effectiveness and safety have been internationally recognized. However, systemic bioavailability is low, and second-generation INC drugs currently in use further reduce system bioavailability (<1%) compared to older INC and oral medications, limiting systemic effects. Studies have found that AR is a local response to systemic inflammation. Systemic inflammation keeps the body in a state of sensitization, which is easy to be triggered by allergens again, leading to repeated diseases. Therefore, the lack of efficacy of the nasal hormone system limits the effectiveness of relapse control. The best treatment for AR should be systemic and local. Effective treatment of the underlying inflammation is critical to control the systemic manifestations of AR. In the acute phase of AR, systemic symptoms such as drowsiness, fatigue, and cognitive decline are usually the complaints of allergy patients, which will reduce their quality of life and cause mental and psychological diseases such as chronic fatigue syndrome and depression.

The comprehensive regulation of multi-target and multi-link regulation is the characteristic of traditional Chinese medicine (TCM). A Meta-analysis showed that Chinese medicine was better than western medicine in controlling the recurrence rate of allergic rhinitis in children. In more than two decades of clinical application and animal experiments, it has been confirmed that MYN has the advantage of controlling AR symptoms. In addition, it also has been observed the effect MYN has on preventing the recurrence of AR in long-term clinical application. However, high-quality, evidence-based evidence needs to support the evaluation of clinical efficacy on recurrence and safety.

**Modified Yupingfeng nasal spray(MYN)** belongs to the Hospital of Chengdu University of Traditional Chinese Medicine prescription, with the specification of 10ml, and its main drugs are composed of Astragalus mongholicus Bunge, Saposhnikovia divaricata, Atractylodes macrocephala Koidz, Etc. It has the effect of beneficial qi to solidify the external, tonify the lung and to invigorate the spleen, dispelling wind and removing evil, and removing qi and blood stasis，It has a good effect on the symptoms of allergic rhinitis such as nasal itching, sneezing, runny nose and nasal congestion.

**II. Research Purpose:**

This study provides high-quality, evidence-based evidence for MYN in the treatment of persistent moderate-severe AR(Qi deficiency and blood stasis syndrome，QDBS) and its efficacy and safety in relapse control. To investigate the possible action mechanism of MYN in the treatment of persistent moderate-severe AR and to provide a clinical basis for further confirmation of the mechanism of action. To clarify the best application course, applicable syndromes, and applicable scope of MYN in the treatment of persistent moderate and severe AR，To supplement the scientific connotation of the clinical pathway of TCM nasal spray in the treatment of persistent moderate-severe AR, and to establish new clinical use specifications of TCM nasal spray.

**III、What do you need to do if you take part in the study?**

1. Before you are enrolled in the study, you will undergo the following tests to determine whether you can participate: Your doctor will ask and record your medical history, clinical signs, and symptoms, current, and past medications. And you'll get a skin prick test(SPT) for allergens, as well as safety indicators: vital signs, routine blood and urine tests, liver and kidney function tests, pregnancy tests, electrocardiogram.

2. If you meet the inclusion criteria above, you will follow the following steps to conduct the study: The experiment was divided into two groups: group A and B. an independent clinical statistician will use SAS 9.4 statistical software to generate a random sequence and every patient will be randomly assigned to receive intervention either in group A or group B. Neither you nor your doctor can know and choose treatment in advance, Among them：

Group A： MYN is produced and packed by the Pharmacy Department of Hospital of Chengdu University of Traditional Chinese Medicine (unlisted). MYN will be administered intranasal, spray two pumps/nostril twice a day. The drug will not be administered once the patient meets the clinical control standard and continues for two weeks; the drug will not be administered. The shortest treatment time is not less than four weeks, and the longest treatment time is not more than three months.

Group B： Mometasone Furoate Aqueous nasal spray(Nasonex) is produced and packed by Schering-Plough Labo N.V.（drug batch number: H20140100）. Mometasone Furoate Aqueous nasal spray will be administered intranasal, spray two pumps/nostril, once a day. The drug will not be administered once the patient meets the clinical control standard and continues for two weeks. The shortest treatment time is not less than four weeks, and the longest treatment time is not more than three months.

**How to use the nasal spray：** You should clean your nasal cavity before using the nasal spray. You can also use seawater nasal spray to clean your nasal cavity. After thoroughly shaking the medicine bottle, spray the medicine into the air 1 to 2 times. The right thumb is held on the bottom of the bottle, the index finger and middle finger are placed on both sides of the nozzle, and the nozzle is clamped. With your head in a natural position, place the nozzle of the nasal spray in front of your left nostril with your right hand, pointing toward your outer canthus (outer corner of your eye), keeping the bottle more or less upright, not over tilted. Press while gently inhaling through your nose and exhaling through your mouth. Repeat with the nasal spray on your left hand. After the nasal spray is used, clean the nozzle, close the cap and place the bottle vertically.

**Clinical control standard**: After patients start treatment, the RCAT scale will be tested every two weeks for each patient. If the RCAT score is >21, it means that AR is well controlled (25 of which is fully controlled); if the ARCT score is ≤ 21, it means that AR is not controlled.

3. Patients will be tested for SPT the day before enrollment, and the following indicators will be observed on the visit day and when the clinical control standards are reached:

（1）Vital signs, ECG, and laboratory tests (liver and kidney function, blood and urine routine examination) will be compared before and after interventions and assess the safety of the intervention when if AR is controlled.

（2）Visual Analogue Scale (VAS), Total Nasal Symptom Scores (TNSS), Rhinoconjunctivitis Quality of Life Questionnaire (RQLQ), Total Ocular Symptom Score (TOSS), TCM Syndrome Score of Spleen Qi Deficiency and Lung Qi Deficiency, Classification and Determination Scale of Constitution of TCM.

（3）For a subgroup of mechanism participants, the epithelial cells of the nasal mucosa were extracted with a nasal brush. The mRNA and protein expression levels of TLR4, MyD88, and TRIF from the nasal mucosa will be measured respectively by qRT-PCR and western blot. The protein expression levels of HO-1, TLR2 will be measured by Western blot. The nasal lavage fluid concentrations of IL4, IFNγ, TNF-α, NF-κΒ, IL-6, and IL-12 will be measured by ELISA. The endogenous carbon monoxide in plasma will be measured by colorimetry. PAF will be measured by quantitative real-time PCR(qRT-PCR). The expressions of TLR4, CD80, CD86, CCR6, and CCR7 on the surface of DCS will be detected by flow cytometry(FCM).

4. If you relapse during follow-up, your physician will contact you by phone, and inquiry Visual Analogue Scale (VAS), Total Nasal Symptom Scores (TNSS), Rhinoconjunctivitis Quality of Life Questionnaire (RQLQ), Total Ocular Symptom Score (TOSS), TCM Syndrome Score of Spleen Qi Deficiency and Lung Qi Deficiency, Classification and Determination Scale of Constitution of TCM.

5. Other matters that need your cooperation

It would help if you visited the hospital according to the doctor's appointment with you. Your follow-up is necessary because your doctor will determine whether the treatment you are receiving is working and provide timely guidance.

You will receive the test drugs after enrollment. Please follow the doctor's instructions and fill in your medication record in the APP timely and objectively.

You must return all used medications and their packaging at each visit and bring any other medications you are taking, including medicines you continue to take if you have other comorbidities.

You shall not give the experimental drug to anyone else and shall not use it for any other purpose.

It would help if you did not use any other treatment for allergic rhinitis during the study period. If you need additional treatment, please get in touch with your doctor.

**IV. Possible benefits of participating in the study**

The medicine used in this study may improve your condition and prevent recurrence; However, this still requires further verification, and there is no guarantee that it will work for you. The information obtained from this study may help doctors and researchers further identify standardized clinical studies of MYN for the prevention and treatment of allergic rhinitis better to apply it to other patients with similar diseases.

**V. Possible risks, discomfort, and inconveniences of participating in the study**

Although previous studies have shown that the safety of this drug is good, all therapeutic medicines may have side effects. Your condition may improve, stay the same, or worsen during the study period. If you have any discomfort, new changes in your state, or any unexpected situation during the study, no matter whether it is related to medication, you should inform the doctor in time, and the doctor will make a judgment and medical treatment for you.

During the study period, you need to visit the hospital on time for follow-up and some tests, which may cause trouble or inconvenience. Therefore, we will compensate you financially for each visit.

**VI. Is it necessary to participate in and complete this study?**

Your participation in this study is entirely voluntary. If you do not wish, you can refuse to participate, which will not negatively impact your current or future medical care. Even after you have agreed to participate, you may change your mind at any time and tell the investigator that you are withdrawing from the study. Your withdrawal will not affect your access to regular medical care.

**VII. Other treatments you can choose besides participating in this study**

Your study physician will discuss with you the other treatment options currently available for your condition, including the associated risks and benefits. Patients with allergic rhinitis may also be treated with second-generation oral antihistamines, which act quickly and for a long time but do not have a good effect on nasal congestion and have the side effect of rare cardiotoxicity.

**VIII. Related expenses**

Fees for all of your examinations (Allergen skin prick test, blood, and urine routine test, liver and kidney function test, pregnancy test, electrocardiogram; Ho-1, TLR2, TLR4, MyD88, TRIF protein levels, TLR4, CD80, CD86, MyD88, TRIF gene levels in nasal mucosal epithelial cells; Il-4, IFNγ, TNF-α, NF-κB, IL-6, IL-12, IL-13 and IL-25 in the washing solution of the nasal cavity; Serum IL-4, quantitative, PAF Level, DCs Surface TLR4, CD80, CD86, CCR6, CCR7), outpatient registration and experimental drugs related to the study are free.

If an adverse event occurs during a clinical trial, a committee of medical experts will determine whether it is related to the treatment. In case of adverse reactions and injuries caused by diagnostic tests and treatment required by research drugs or protocols, the research group will pay the related treatment costs and the corresponding economic compensation according to *China's Quality Management Practice for Drug Clinical Trials.*

If you have other diseases simultaneously, the treatment and examination will not be free of charge.

**IX. Is personal information confidential?**

Your medical records will be kept entirely at the hospital where you are treated. Your doctor will record the results of the laboratory tests on your medical history. The investigator, ethics committee, and program management will be allowed access to your medical records. Any public report on the results of this study will not disclose your identity. We will make every effort to protect the privacy of your medical data to the extent permitted by law.

**X.** **You may voluntarily choose to participate in the study or withdraw from the study during the course of the study**

Whether or not to participate in the study is entirely up to you. You may refuse to participate in the study or withdraw from the study at any time during the study without affecting your medical treatment and benefits.

In your best interest, your physician or investigator may discontinue your continued participation in the study at any time during the study. If you withdraw from the study for any reason, you may be asked about your use of the investigational drug. You may also be required to have laboratory tests and a physical examination if your doctor deems it necessary.

Your doctor will notify you of any significant events or information related to the subjects that may affect your willingness to continue to participate in the study.

**XI、How do you get more information?**

If you have any questions, suggestions, or complaints about this research, please discuss with the research team in time. For contact information, see the signature page. If you feel inconvenient to communicate with the research team, you can contact the Medical Ethics Committee of The Hospital of Chengdu University of Traditional Chinese Medicine (028-87783142, 028-87783139) for consultation or complaint.

**Informed Consent · Consent signature page**

**Project name：** Efficacy of Ke-Min-Qi-Dan nasal spray in controlling recurrence of persistent moderate-severe allergic rhinitis: a multicenter, open-label, randomized, and parallel controlled trial

1、I have read the above introduction to this study and have had the opportunity to discuss and ask questions about this study with doctors. All my questions have been satisfactorily answered.

2 、I am aware of the possible risks and benefits of participating in this study. I understand that my participation in the survey is voluntary; I confirm that I have had enough time to consider it and understand that:

(1)I can always consult my doctor for more information.

(2)I can withdraw from the study at any time without discrimination or retaliation, and my medical treatment and rights will not be affected.

(3)I also know that if I drop out of the study, especially for medical reasons, I should inform my doctor of the changes in my condition and complete the corresponding physical and chemical examination, which will significantly benefit the whole study.

(4)If I need to take any other medication due to a change in my condition, I will seek advice from my doctor before or tell my doctor truthfully afterward.

(5)I grant my research materials to the researchers, sponsors' representatives, and the Ethics committee.

(6)I will be given a signed and dated copy of the informed consent.

In the end, I agreed to participate in the study and promised to follow the doctor's advice as much as possible.

Signature of the patient: date

Phone number：

I confirm that I have explained to the patient the details of the trial, including his rights and the possible benefits and risks, and have given him/her a copy of the signed informed consent form.

Signature of doctor：	date

Phone number：
